# Nonlinear strict distance and similarity measures for intuitionistic fuzzy sets with applications to pattern classification and medical diagnosis

**DOI:** 10.1038/s41598-023-40817-y

**Published:** 2023-08-25

**Authors:** Xinxing Wu, Huan Tang, Zhiyi Zhu, Lantian Liu, Guanrong Chen, Miin-Shen Yang

**Affiliations:** 1 School of Mathematics and Statistics, Guizhou University of Finance and Economics, Guiyang, 550025 Guizhou China; 2 School of Sciences, Southwest Petroleum University, Chengdu, 610500 Sichuan China; 3 Department of Electrical Engineering, City University of Hong Kong, Hong Kong, Hong Kong SAR China; 4 Department of Applied Mathematics, Chung Yuan Christian University, Taoyuan, 32023 Taiwan

**Keywords:** Engineering, Mathematics and computing

## Abstract

In this paper, we propose a new type of nonlinear strict distance and similarity measures for intuitionistic fuzzy sets (IFSs). Our proposed methods not only have good properties, but also improve the drawbacks proposed by Mahanta and Panda (Int J Intell Syst 36(2):615–627, 2021) in which, for example, their distance value of $d_{_{\textrm{MP}}}(\langle \mu , \nu \rangle , \langle 0, 0\rangle )$ is always equal to the maximum value 1 for any intuitionistic fuzzy number $\langle \mu , \nu \rangle \ne \langle 0, 0\rangle$. To resolve these problems in Mahanta and Panda (Int J Intell Syst 36(2):615–627, 2021), we establish a nonlinear parametric distance measure for IFSs and prove that it satisfies the axiomatic definition of strict intuitionistic fuzzy distances and preserves all advantages of distance measures. In particular, our proposed distance measure can effectively distinguish different IFSs with high hesitancy. Meanwhile, we obtain that the dual similarity measure and the induced entropy of our proposed distance measure satisfy the axiomatic definitions of strict intuitionistic fuzzy similarity measure and intuitionistic fuzzy entropy. Finally, we apply our proposed distance and similarity measures to pattern classification, decision making on the choice of a proper antivirus face mask for COVID-19, and medical diagnosis problems, to illustrate the effectiveness of the new methods.

## Introduction

Zadeh^1^ introduced the concept of fuzzy sets (FSs) by using a function from the universe of discourse to [0, 1], which was called the membership function, to describe the importance of an element in the universe of discourse. Zadeh’s fuzzy set theory had been applied in different areas^2–4^. However, FSs can only deal with the situation containing two opposite responses. It fails to deal with the situation with the hesitant/neutral state of “this and also that”. As a remedy, Atanassov^5^ generalized Zadeh’s fuzzy set by proposing the concept of intuitionistic fuzzy sets (IFSs), characterized by a membership function and a non-membership function meeting the condition that their sum at every point is less than or equal to 1. Since then, IFSs have been widely applied to various fields, such as multiple attribute decision making (MADM)^6–11^, medical diagnosis^12–15^, similarity with pattern recognition^16–19^, and cluster analysis^16,20–22^.

Being a pair of dual concepts, the intuitionistic fuzzy (IF) distance measure (IFDisM) and the IF similarity measure (IFSimM) are useful for measuring the differences of IFSs under IF situations. The axiomatic definitions of IFDisMs and IFSimMs were first given by Wang and Xin^23^. Szmidt^24^ considered IFDisMs and IFSimMs and divided them into two types of IFSs according to 2-dimensional (2D) and 3-dimensional (3D) representations. However, Wu et al.^25^ used some examples to show that many existing 3D IFDisMs and IFSimMs, including Euclidean DisM and SimM^24^, Minkowski DisM and SimM^26,27^, do not satisfy the axiomatic definitions of IFDisMs and IFSimMs. Burillo and Bustince^28^ introduced the 2D Hamming IFDisM. Grzegorzewski^29^ and Hung and Yang^30^ presented some new IFSimMs and IFDisMs based on Hausdorff metric. Wang and Xin^23^ obtained a new IFDisM by combining the 2D Hamming IFDisM^28^ and the 2D Hausdorff IFDisM^29^. Hwang and Yang^31^ introduced a new IFSimM via lower, upper and middle fuzzy sets. Xiao^32^ obtained a 3D IFDisM based on Jensen-Shannon divergence and illustrated that it is better than the IFDisMs in^33–36^. However, Wu et al.^37^ showed some examples to illustrate that Xiao’s DisM does not satisfy the axiomatic definition of IFDisMs. Meanwhile, Wu et al.^37^ first introduced the concepts of strict IFDisM, and then obtained a new strict IFDisM via Jensen-Shannon divergence to more effectively compare and distinguish IFNs and IFSs.

To accurately distinguish different IFSs with high indeterminacy degrees, Mahanta and Panda^38^ developed a new nonlinear 2D IFDisM. However, their DisM $d_{_{\textrm{MP}}}$ has the following two drawbacks: (1) the value of $d_{_{\textrm{MP}}}(\langle \mu , \nu \rangle , \langle 0, 0\rangle )$ is always equal to the maximum value 1 for any IFN $\langle \mu , \nu \rangle \ne \langle 0, 0\rangle$; (2) $d_{_{\textrm{MP}}}(\langle \mu , 0\rangle , \langle 0, \nu \rangle )=1$ holds for all $\mu , \nu \in (0, 1]$. These are unreasonable results. To overcome the above two drawbacks, we construct a nonlinear parametric IFDisM and show that it is a strict IFDisM, which preserves all advantages of Mahanta and Panda’s DisM^38^. Moreover, we prove that the dual SimM and the induced entropy of our proposed IFDisM satisfy the axiomatic definitions of IFSimM and IF entropy. Additionally, we apply the proposed IFDisM and IFSimM to pattern classification, decision making for the choice of a proper antivirus face mask for COVID-19, and medical diagnosis, to illustrate the effectiveness of the new methods.

## Preliminaries

### Intuitionistic fuzzy set (IFS)

#### Definition 2.1

(^39^Definition 1.1) An *intuitionistic fuzzy set* (IFS) *I* in $\Xi$ is defined as an object in the following form

1 $$\begin{aligned} I=\left\{ \langle \vartheta , \mu _{_{I}}(\vartheta ), \nu _{_{I}}(\vartheta )\rangle \mid \vartheta \in \Xi \right\} , \end{aligned}$$

where the functions $\mu _{_{I}}: \Xi \rightarrow [0,1]$ and $\nu _{_{I}}: \Xi \rightarrow [0,1]$ are the *degree of membership* and the *degree of non-membership* of an element $\vartheta \in \Xi$ to the set *I*, respectively; and for any $\vartheta \in \Xi$,

2 $$\begin{aligned} \mu _{_{I}}(\vartheta )+\nu _{_{I}}(\vartheta )\le 1. \end{aligned}$$

Let $\textrm{IFS}(\Xi )$ denote the set of all IFSs in $\Xi$. For $I\in \textrm{IFS}(\Xi )$, the *indeterminacy degree* $\pi _{_{I}}(\vartheta )$ of an element $\vartheta$ belonging to *I* is defined by $\pi _{_I}(\vartheta )=1-\mu _{_I}(\vartheta )-\nu _{_I}(\vartheta )$. The pair $\langle \mu _{_I}(\vartheta ), \nu _{_I}(\vartheta )\rangle$ is called an * intuitionistic fuzzy value* (IFV) or an *intuitionistic fuzzy number* (IFN) by Xu^10^. For convenience, we use $\alpha =\langle \mu _{\alpha }, \nu _{\alpha }\rangle$ to represent an IFN $\alpha$, which satisfies $\mu _{\alpha }\in [0, 1]$, $\nu _{\alpha }\in [0, 1]$, and $0\le \mu _{\alpha }+\nu _{\alpha }\le 1$. Let $\Theta$ be the set of all IFNs, i.e., $\Theta =\{\langle \mu , \nu \rangle \in [0, 1]^{2} \mid \mu +\nu \le 1\}$. For $\alpha =\langle \mu _{\alpha }, \nu _{\alpha }\rangle \in \Theta$, the *complement* $\alpha ^{\complement }$ of $\alpha$ is $\alpha ^{\complement }=\langle \nu _{\alpha }, \mu _{\alpha }\rangle$.

Atanassov’s order ‘$\subset$’^39^, defined by the condition that $\alpha \subset \beta$ if and only if $\alpha \cap \beta =\alpha$, is a partial order on $\Theta$. Clearly, $\alpha \subset \beta$ if and only if $\mu _{\alpha }\le \mu _{\beta }$ and $\nu _{\alpha }\ge \nu _{\beta }$. The order ‘$\subsetneqq$’ on $\Theta$ is defined by the condition that $\alpha \subsetneqq \beta$ if and only if $\alpha \subset \beta$ and $\alpha \ne \beta$.

### Similarity/distance measures for IFSs

#### Definition 2.2

^10,26^A mapping $\textbf{S}: \Theta \times \Theta \longrightarrow [0, 1]$ is called an *intuitionistic fuzzy similarity measure* (IFSimM) on $\Theta$ if it satisfies the following conditions: for any $\alpha _1$, $\alpha _2$, $\alpha _3\in \Theta$,

1. $$0\le \textbf{S}(\alpha _1, \alpha _2)\le 1$$.
2. $\textbf{S}(\alpha _1, \alpha _2)=1$ if and only if $\alpha _1=\alpha _2$.
3. $$\textbf{S}(\alpha _1, \alpha _2)=\textbf{S}(\alpha _2, \alpha _1)$$.
4. If $\alpha _1\subset \alpha _2\subset \alpha _3$, then $\textbf{S}(\alpha _1, \alpha _3)\le \textbf{S}(\alpha _1, \alpha _2)$ and $\textbf{S}(\alpha _1, \alpha _3) \le \textbf{S}(\alpha _2, \alpha _3)$.

#### Definition 2.3

^10,26^A mapping $\textbf{S}: \textrm{IFS}(\Xi )\times \textrm{IFS}(\Xi ) \longrightarrow [0, 1]$ is called an *IFSimM* on $\textrm{IFS}(\Xi )$ if it satisfies the following conditions: for any $I_1$, $I_2$, $I_3\in \textrm{IFS}(\Xi )$,

1. $$0\le \textbf{S}(I_1, I_2)\le 1$$.
2. $\textbf{S}(I_1, I_2)=1$ if and only if $I_1=I_2$.
3. $$\textbf{S}(I_1, I_2)=\textbf{S}(I_2, I_1)$$.
4. If $I_1\subset I_2\subset I_3$, then $\textbf{S}(I_1, I_3)\le \textbf{S}(I_1, I_2)$ and $\textbf{S}(I_1, I_3)$ $\le \textbf{S}(I_2, I_3)$.

To more effectively compare and distinguish IFNs and IFSs, the concept of strict intuitionistic fuzzy similarity/distance measures was introduced by Wu et al.^37^ as follows.

#### Definition 2.4

^37^A mapping $\textbf{S}: \Theta \times \Theta \longrightarrow [0, 1]$ is called a *strict IFSimM* on $\Theta$ if, for any $\alpha _1$, $\alpha _2$, $\alpha _3\in \Theta$, it satisfies (Sl)–(S3) in Definition 2.2 and (S4$^{\prime }$) and (S5) described by

(S4) (S4$^{\prime }$) (Strict distinctiveness) If $\alpha _1\subsetneqq \alpha _2\subsetneqq \alpha _3$, then $\textbf{S}(\alpha _1, \alpha _3)< \textbf{S}(\alpha _1, \alpha _2)$ and $\textbf{S}(\alpha _1, \alpha _3) < \textbf{S}(\alpha _2, \alpha _3)$.
(S5) (Extreme dissimilarity on endpoints) $\textbf{S}(\alpha _1, \alpha _2)=0$ if and only if ($\alpha _1=\langle 0, 1\rangle$ and $\alpha _2=\langle 1, 0 \rangle$) or ($\alpha _1=\langle 1, 0\rangle$ and $\alpha _2=\langle 0, 1 \rangle$).

As pointed out by Wu et al.^37^, (1) Property (S4$^{\prime }$) indicates that the similarity measure $\textbf{S}$ can strictly distinguish every pair of different IFVs under the Atanassov-order ‘$\subset$’; (2) Property (S5) indicates that it is extremely unsimilar (similarity measure is zero) for a pair of IFVs depending only on two endpoints.

#### Definition 2.5

^37^A mapping $\textbf{S}: \textrm{IFS}(\Xi )\times \textrm{IFS}(\Xi ) \longrightarrow [0, 1]$ is called a *strict IFSimM* on $\textrm{IFS}(\Xi )$ if, for any $I_1$, $I_2$, $I_3\in \textrm{IFS}(\Xi )$, it satisfies (Sl)–(S3) in Definition 2.3 and (S4$^{\prime }$) and (S5) described by

(S4$$^{\prime }$$)
If $I_1\subsetneqq I_2\subsetneqq I_3$, then $\textbf{S}(I_1, I_3)< \textbf{S}(I_1, I_2)$ and $\textbf{S}(I_1, I_3)$ $< \textbf{S}(I_2, I_3)$.
(S5): $\textbf{S}(I_1, I_2)=0$ if and only if, for any $\vartheta \in \Xi$, ($I_1(\vartheta )=\langle 0, 1\rangle$ and $I_2(\vartheta )=\langle 1, 0 \rangle$) or ($I_1(\vartheta )=\langle 1, 0\rangle$ and $I_2(\vartheta )=\langle 0, 1 \rangle$).

#### *Remark* 1

Property (S5) can be equivalently expressed as that $\textbf{S}(I_1, I_2)=0$ if and only if $I_1$ is a crisp set and $I_{1}=I_{2}^{\complement }$.

Dually, a mapping $d: \textrm{IFS}(\Xi )\times \textrm{IFS}(\Xi ) \longrightarrow [0, 1]$ is called a *strict IFDisM* on $\textrm{IFS}(\Xi )$ if the mapping $\textbf{S}(I_1, I_2)=1-d(I_1, I_2)$ is a strict IFSimM on $\textrm{IFS}(\Xi )$.

### Entropy measure for IFSs

Entropy is an important information measure. Szmidt and Kacprzyk^15^ gave the axiomatic definitions of entropy measures for IFSs as follows:

#### Definition 2.6

^15^A mapping $E: \Theta \longrightarrow [0, 1]$ is called an *intuitionistic fuzzy entropy measure* (IFEM) on $\Theta$ if it satisfies the following conditions: for any $\alpha$, $\beta \in \Theta$,

1. $E(\alpha )=0$ if and only if $\alpha =\langle 1, 0\rangle$ or $\alpha =\langle 0, 1\rangle$.
2. $E(\alpha )=1$ if and only if $\mu _{\alpha }=\nu _{\alpha }$.
3. $$E(\alpha )=E(\alpha ^{\complement })$$.
4. $E(\alpha )\le E(\beta )$ whenever it holds either $\mu _{\alpha }\le \mu _{\beta } \le \nu _{\beta } \le \nu _{\alpha }$ or $\mu _{\alpha }\ge \mu _{\beta } \ge \nu _{\beta } \ge \nu _{\alpha }$.

#### Definition 2.7

^15^A mapping $E: \textrm{IFS}(\Xi )\longrightarrow [0, 1]$ is called an *IFEM* on $\textrm{IFS}(\Xi )$ if it satisfies the following conditions: for any $I_1$, $I_2 \in \textrm{IFS}(\Xi )$,

1. $E(I_1)=0$ if and only if $I_1$ is a crisp sets.
2. $E(I_1)=1$ if and only if, for any $\vartheta \in \Xi$, $\mu _{I_1}(\vartheta )=\nu _{I_1}(\vartheta )$.
3. $$E(I_1)=E(I_1^{\complement })$$.
4. $E(I_1)\le E(I_2)$ if, for any $\vartheta \in \Xi$, it holds either $\mu _{I_1}(\vartheta )\le \mu _{I_2}(\vartheta )\le \nu _{I_2}(\vartheta ) \le \nu _{I_1}(\vartheta )$ or $\mu _{I_1}(\vartheta )\ge \mu _{I_2}(\vartheta )\ge \nu _{I_2}(\vartheta ) \ge \nu _{I_1}(\vartheta )$.

## The proposed nonlinear strict distance, similarity and entropy measures for IFSs

After we investigate the distance measure for IFSs proposed by Mahanta and Panda^38^, we find that Mahanta and Panda’s^38^ distance gave serious drawbacks. We present these drawbacks in next subsection.

### The drawbacks of distance measure of Mahanta and Panda^38^

Let $\Xi =\{\vartheta _1, \vartheta _2, \ldots , \vartheta _{\ell }\}$ be a finite UOD and $I_1=\Big \{\frac{\langle \mu _{I_1}(\vartheta _{j}), \nu _{I_1}(\vartheta _{j})\rangle }{\vartheta _j} \mid 1\le j\le \ell \Big \}$ and $I_2= \Big \{\frac{\langle \mu _{I_2}(\vartheta _{j}), \nu _{I_2}(\vartheta _{j})\rangle }{\vartheta _j} \mid 1\le j\le \ell \Big \}$ be two IFSs on $\Xi$. To effectively distinguish IFSs with high degrees of hesitancy, Mahanta and Panda^38^ recently introduced a 2D IFDisM $d_{_{\textrm{MP}}}$ as follows:

3 $$\begin{aligned} d_{_{\textrm{MP}}}(I_1, I_2)=\frac{1}{\ell }\sum _{j=1}^{\ell } \frac{|\mu _{I_1}(\vartheta _{j})-\mu _{I_2}(\vartheta _{j})| +|\nu _{I_1}(\vartheta _{j})-\nu _{I_2}(\vartheta _{j})|}{\mu _{I_1}(\vartheta _{j})+\mu _{I_2}(\vartheta _{j}) +\nu _{I_1}(\vartheta _{j})+\nu _{I_2}(\vartheta _{j})}. \end{aligned}$$

This subsection uses two examples to show that their IFDisM $d_{_{\textrm{MP}}}$^38^ has the following two drawbacks: (1) the distance from all IFVs except $\langle 0, 0 \rangle$ to $\langle 0, 0 \rangle$ obtained by the distance formula $d_{_{\textrm{MP}}}$ is equal to the maximum value 1, i.e., $d_{_{\textrm{MP}}} (\langle 0, 0\rangle , \alpha )=1$ holds for all $\alpha \in \Theta \backslash \{\langle 0, 0\rangle \}$; (2) $d_{_{\textrm{MP}}}(\langle \mu , 0\rangle , \langle 0, \nu \rangle )=1$ holds for all $\mu , \nu \in (0, 1]$. These are unreasonable results.

#### *Example* 3.1

Let $\Xi =\{\vartheta \}$ and $I_1=\left\{ \frac{\langle 0, 0\rangle }{\vartheta }\right\} \in \textrm{IFS}(\Xi )$. For any $I_2=\left\{ \frac{\langle \mu , \nu \rangle }{\vartheta }\right\} \in \textrm{IFS}(\Xi )$ with $I_2\ne I_1$, by direct calculation and Eq. (3), we have $d_{_{\textrm{MP}}}(I_1, I_2)= \frac{|\mu -0|+|\nu -0|}{\mu +0+\nu +0}=1$. This is obviously an unreasonable result, since all points except $\left\{ \frac{\langle 0, 0 \rangle }{\vartheta }\right\}$ to $\left\{ \frac{\langle 0, 0 \rangle }{\vartheta }\right\}$ is equal to the maximum value 1.

#### *Example* 3.2

Let $\Xi =\{\vartheta \}$, $I_1^{\prime }=\left\{ \frac{\langle \mu , 0\rangle }{\vartheta }\right\} \in \textrm{IFS}(\Xi )$, and $I_2^{\prime }=\left\{ \frac{\langle 0, \nu \rangle }{\vartheta }\right\} \in \textrm{IFS}(\Xi )$. By direct calculation and Eq. (3), we have that, for $0<\mu , \nu \le 1$, $d_{_{\textrm{MP}}}(I_1^{\prime }, I_2^{\prime })=\frac{|\mu -0|+|0-\nu |}{\mu +0+0+\nu }=1$, which is also an unreasonable result.

To overcome the drawbacks of Mahanta and Panda’s distance measure mentioned above, we propose a new nonlinear strict distance measure for IFNs and IFSs in next subsection, which is proved to satisfy the axiomatic definition of IFDisM.

### A new parametric distance on $\Theta$

We define a new parametric distance on $\Theta$ by defining the function $d_{\textrm{pd}}^{(\lambda )}: \Theta \times \Theta \longrightarrow \mathbb {R}^{+}$ as follows: for $\alpha =\langle \mu _{\alpha }, \nu _{\alpha }\rangle$ and $\beta = \langle \mu _{\beta }, \nu _{\beta }\rangle \in \Theta$,

4 $$\begin{aligned} d_{\textrm{pd}}^{(\lambda )}(\alpha , \beta )= \frac{|\mu _{\alpha }-\mu _{\beta }|+|\nu _{\alpha }-\nu _{\beta }|}{\mu _{\alpha }+\nu _{\alpha }+\mu _{\beta }+\nu _{\beta }+\lambda } \cdot \frac{2+\lambda }{2}. \end{aligned}$$

#### Lemma 3.1

*Let* $\lambda >0$. *For* $0\le x\le y\le 2$, *the following statements hold*:

1. $$\frac{x}{y+\lambda }\le \frac{2}{2+\lambda }$$;
2. $\frac{x}{y+\lambda }= \frac{2}{2+\lambda }$ *if and only if* $x=y=2$.

#### *Proof*

1. From $0\le x\le y$, it follows that $\frac{x}{y+\lambda } \le \frac{y}{y+\lambda }$. This, together with $0\le y\le 2$, implies that $\frac{y}{y+\lambda }\le \frac{2}{2+\lambda }$.
2. It follows directly from the proof of (1).

$$\square $$

#### Proposition 3.1

$$0\le d_{_{\textrm{pd}}}^{(\lambda )}(\alpha , \beta ) \le 1$$.

#### *Proof*

Note that $0\le |\mu _{\alpha }-\mu _{\beta }|+|\nu _{\alpha }-\nu _{\beta }| \le \mu _{\alpha }+\nu _{\alpha }+\mu _{\beta }+\nu _{\beta }\le 2$. By  Lemma 3.1, it follows that $d_{_{\textrm{pd}}}^{(\lambda )}(\alpha , \beta )= \frac{|\mu _{\alpha }-\mu _{\beta }|+|\nu _{\alpha }-\nu _{\beta }|}{\mu _{\alpha }+\nu _{\alpha }+\mu _{\beta }+\nu _{\beta }+\lambda } \cdot \frac{2+\lambda }{2}\le \frac{2}{2+\lambda } \cdot \frac{2+\lambda }{2}=1$. $\square$

#### Proposition 3.2

$$d_{_{\textrm{pd}}}^{(\lambda )}(\alpha , \beta )= d_{_{\textrm{pd}}}^{(\lambda )}(\beta , \alpha )$$.

#### *Proof*

It follows directly from Eq. (4). $\square$

#### Proposition 3.3

$d_{_{\textrm{pd}}}^{(\lambda )}(\alpha , \beta )=0$ *if and only if* $\alpha =\beta$.

#### *Proof*

Note that $\lambda >0$, and by Eq. (4), it follows that $d_{_{\textrm{pd}}}^{(\lambda )}(\alpha , \beta )=0$ if and only if $|\mu _{\alpha }-\mu _{\beta }|+|\nu _{\alpha }-\nu _{\beta }|=0$ if and only if $\mu _{\alpha }=\mu _{\beta }$ and $\nu _{\alpha }=\nu _{\beta }$. $\square$

#### Proposition 3.4

$d_{_{\textrm{pd}}}^{(\lambda )}(\alpha , \beta )=1$ *if and only if* {$\alpha =\langle 0, 1 \rangle$ *and* $\beta =\langle 1, 0 \rangle$}, or {$\alpha =\langle 1, 0 \rangle$ *and* $\beta =\langle 0, 1 \rangle$}.

#### *Proof*

*Sufficiency.* By direct calculation and Eq. (4), it follows that $d_{_{\textrm{pd}}}^{(\lambda )}(\langle 0, 1\rangle , \langle 1, 0\rangle ) =d_{_{\textrm{pd}}}^{(\lambda )}(\langle 1, 0\rangle , \langle 0, 1\rangle )=1$.

*Necessity.* By Lemma 3.1 (2), it follows that $d_{_{\textrm{pd}}}^{(\lambda )}(\alpha , \beta )=1$ if and only if $|\mu _{\alpha }-\mu _{\beta }|+|\nu _{\alpha }-\nu _{\beta }|=2$ implying that $|\mu _{\alpha }-\mu _{\beta }|=1$ and $|\nu _{\alpha }-\nu _{\beta }|=1$. And thus ($\alpha =\langle 0, 1 \rangle$ and $\beta =\langle 1, 0 \rangle$) or ($\alpha =\langle 1, 0 \rangle$ and $\beta =\langle 0, 1 \rangle$). $\square$

#### Proposition 3.5

Let $$\alpha $$, $$\beta $$, $$\gamma \in \Theta $$.

1. *If* $\alpha \subset \beta \subset \gamma$, *then* $d_{_{\textrm{pd}}}^{(\lambda )}(\alpha , \gamma ) \ge d_{_{\textrm{pd}}}^{(\lambda )}(\alpha , \beta )$ *and* $d_{_{\textrm{pd}}}^{(\lambda )}(\alpha , \gamma )\ge d_{_{\textrm{pd}}}^{(\lambda )}(\beta , \gamma )$.
2. *If* $\alpha \subsetneqq \beta \subsetneqq \gamma$, then $d_{_{\textrm{pd}}}^{(\lambda )}(\alpha , \gamma )> d_{_{\textrm{pd}}}^{(\lambda )}(\alpha , \beta )$ *and* $d_{_{\textrm{pd}}}^{(\lambda )}(\alpha , \gamma )> d_{_{\textrm{pd}}}^{(\lambda )}(\beta , \gamma )$.

#### *Proof*

1. Fix an IFV $\alpha =\langle \mu _{\alpha }, \nu _{\alpha } \rangle \in \Theta$. For any $\tilde{\alpha }=\langle \mu , \nu \rangle \in \Theta$ with $\tilde{\alpha }\supset \alpha$, define a function
  $$\begin{aligned} \zeta (\mu , \nu ) =d_{_{\textrm{pd}}}^{(\lambda )}(\alpha , \tilde{\alpha })=\frac{\mu -\mu _{\alpha } +\nu _{\alpha }-\nu }{\mu _{\alpha }+\nu _{\alpha }+\mu +\nu +\lambda } \cdot \frac{2+\lambda }{2}. \end{aligned}$$
  By direct calculation, we have
  5 $$\begin{aligned} \frac{\partial \zeta }{\partial \mu }=\frac{2\mu _{\alpha } +2\nu +\lambda }{(\mu _{\alpha }+\nu _{\alpha }+\mu +\nu +\lambda )^{2}} \cdot \frac{2+\lambda }{2}> 0, \end{aligned}$$
  and
  6 $$\begin{aligned} \frac{\partial \zeta }{\partial \nu }=\frac{-2\nu _{\alpha } -2\mu -\lambda }{(\mu _{\alpha }+\nu _{\alpha }+\mu +\nu +\lambda )^{2}} \cdot \frac{2+\lambda }{2}< 0. \end{aligned}$$
  This, together with $\alpha \subset \beta \subset \gamma$, i.e., $\mu _{\alpha }\le \mu _{\beta } \le \mu _{\gamma }$ and $\nu _{\alpha } \ge \nu _{\beta } \ge \nu _{\gamma }$, implies that $d_{_{\textrm{pd}}}^{(\lambda )}(\alpha , \beta )=\zeta (\mu _{\beta }, \nu _{\beta }) \le \zeta (\mu _{\gamma }, \nu _{\beta }) \le \zeta (\mu _{\gamma }, \nu _{\gamma }) =d_{_{\textrm{pd}}}^{(\lambda )}(\alpha , \gamma )$. Similarly, it can be verified that $d_{_{\textrm{pd}}}^{(\lambda )}(\alpha , \gamma ) \ge d_{_{\textrm{pd}}}^{(\lambda )}(\beta , \gamma )$.
2. Suppose that, on the contrary, $d_{_{\textrm{pd}}}^{(\lambda )}(\alpha , \gamma ) \ngtr d_{_{\textrm{pd}}}^{(\lambda )}(\alpha , \beta )$ or $d_{_{\textrm{pd}}}^{(\lambda )}(\alpha , \gamma ) \ngtr d_{_{\textrm{pd}}}^{(\lambda )}(\beta , \gamma )$. Without loss of generality, assume that $d_{_{\textrm{pd}}}^{(\lambda )}(\alpha , \gamma ) \ngtr d_{_{\textrm{pd}}}^{(\lambda )}(\alpha , \beta )$. This, together with (1), implies that $d_{_{\textrm{pd}}}^{(\lambda )}(\alpha , \gamma ) = d_{_{\textrm{pd}}}^{(\lambda )}(\alpha , \beta )$. From $\beta \subsetneqq \gamma$, it follows that ($\mu _{\beta }<\mu _{\gamma }$ and $\nu _{\beta }\ge \nu _{\gamma }$) or ($\mu _{\beta }\le \mu _{\gamma }$ and $\nu _{\beta }> \nu _{\gamma }$). Next, we consider the following two cases: 2-1) If $\mu _{\beta }<\mu _{\gamma }$ and $\nu _{\beta }\ge \nu _{\gamma }$, then, by Eqs. (5) and (6), we have $d_{_{\textrm{pd}}}^{(\lambda )}(\alpha , \beta )=\zeta (\mu _{\beta }, \nu _{\beta }) <\zeta (\mu _{\gamma }, \nu _{\beta })\le \zeta (\mu _{\gamma }, \nu _{\gamma }) =d_{_{\textrm{pd}}}^{(\lambda )}(\alpha , \gamma )$, which contradicts with $d_{_{\textrm{pd}}}^{(\lambda )}(\alpha , \gamma ) = d_{_{\textrm{pd}}}^{(\lambda )}(\alpha , \beta )$. 2-2) If $\mu _{\beta }\le \mu _{\gamma }$ and $\nu _{\beta }> \nu _{\gamma }$, then, by Eqs. (5) and (6), we have $d_{_{\textrm{pd}}}^{(\lambda )}(\alpha , \beta )=\zeta (\mu _{\beta }, \nu _{\beta }) <\zeta (\mu _{\beta }, \nu _{\gamma })\le \zeta (\mu _{\gamma }, \nu _{\gamma }) =d_{_{\textrm{pd}}}^{(\lambda )}(\alpha , \gamma )$, which contradicts with $d_{_{\textrm{pd}}}^{(\lambda )}(\alpha , \gamma ) = d_{_{\textrm{pd}}}^{(\lambda )}(\alpha , \beta )$. Therefore, $d_{_{\textrm{pd}}}^{(\lambda )}(\alpha , \gamma )> d_{_{\textrm{pd}}}^{(\lambda )}(\alpha , \beta )$ and $d_{_{\textrm{pd}}}^{(\lambda )}(\alpha , \gamma )> d_{_{\textrm{pd}}}^{(\lambda )}(\beta , \gamma )$.

$$\square $$

Based on the defined parametric distance $d_{\textrm{pd}}^{(\lambda )}$, we can define a similarity measure $\textbf{S}_{\textrm{ps}}(\alpha , \beta )$ on $\Theta$ as follows: for $\alpha =\langle \mu _{\alpha }, \nu _{\alpha }\rangle$ and $\beta = \langle \mu _{\beta }, \nu _{\beta }\rangle \in \Theta$,

7 $$\begin{aligned} \textbf{S}_{_{\textrm{ps}}}(\alpha , \beta )=1-d_{_{\textrm{pd}}}^{(\lambda )} (\alpha , \beta ) \end{aligned}$$

According to Propositions 3.1 and  3.5, we have the following results.

#### Theorem 3.1

1. *The function* $d_{_{\textrm{pd}}}^{(\lambda )}$ *defined by Eq*. (4) *is a strict distance measure on* $\Theta$.
2. *The function* $\textbf{S}_{_{\textrm{ps}}}(\alpha , \beta )$ *defined by Eq*. (7) *is a strict similarity measure on* $\Theta$.

Similarly, we can define a new measure *E* on $\Theta$ based on the parametric distance $d_{_{\textrm{pd}}}^{(\lambda )}$ as follows:

8 $$\begin{aligned} \begin{aligned} E: \Theta&\longrightarrow [0, 1], \\ \alpha&\longmapsto 1-d_{_{\textrm{pd}}}^{(\lambda )} (\alpha , \alpha ^{\complement }), \end{aligned} \end{aligned}$$

#### Theorem 3.2

*Let* $\lambda >0$. *The measure* *E* *defined by Eq*. (8) *is an entropy on* $\Theta$.

#### *Proof*

(E1), (E2), and (E3) follow directly from Propositions 3.3, 3.4 and Eq. (4), respectively.

(E4) For $\alpha$, $\beta \in \Theta$, consider the following two cases:

(E4-1) If $\mu _{\alpha }\le \mu _{\beta } \le \nu _{\beta } \le \nu _{\alpha }$, then $\alpha \subset \beta \subset \beta ^{\complement } \subset \alpha ^{\complement }$. This, together with Proposition 3.5, implies that $E(\alpha )= 1-d_{_{\textrm{pd}}}^{(\lambda )}(\alpha , \alpha ^{\complement }) \le 1-d_{_{\textrm{pd}}}^{(\lambda )}(\alpha , \beta ^{\complement }) \le 1-d_{_{\textrm{pd}}}^{(\lambda )}(\beta , \beta ^{\complement }) =E(\beta )$;

(E4-2) If $\mu _{\alpha }\ge \mu _{\beta } \ge \nu _{\beta } \ge \nu _{\alpha }$, then $\alpha ^{\complement } \subset \beta ^{\complement } \subset \beta \subset \alpha$. This, together with Proposition 3.5, implies that $E(\alpha )= 1-d_{_{\textrm{pd}}}^{(\lambda )}(\alpha , \alpha ^{\complement }) \le 1-d_{_{\textrm{pd}}}^{(\lambda )}(\alpha , \beta ^{\complement }) \le 1-d_{_{\textrm{pd}}}^{(\lambda )}(\beta , \beta ^{\complement }) =E(\beta )$. $\square$

**Figure 1:**
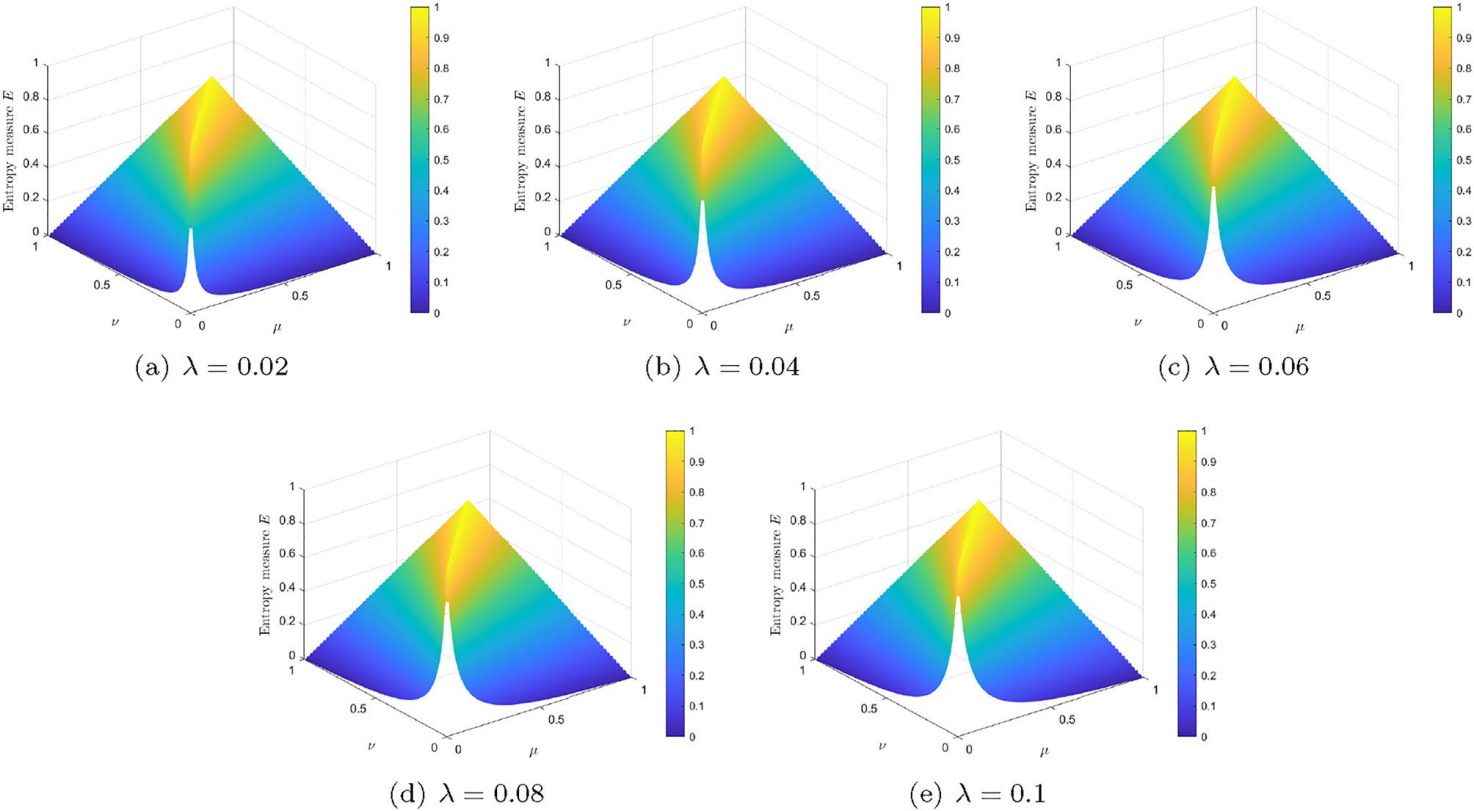
Entropy measure *E* for different values of $\lambda$.

Figure 1 shows the graphs of the entropy measure *E* of Eq. (8) for $\lambda =0.02, 0.04, 0.06, 0.08, 0.1$.

Mahanta and Panda^38^ claimed that the IFDisM $d_{_{\textrm{MP}}}$ can deal adequately with the IF information having high uncertainty, i.e., having low values of membership and nonmembership grades. To close this section, it is shown that our proposed parametric distance $d_{_{\textrm{pd}}}^{(\lambda )}$ can effectively distinguish different IFVs with high hesitancy.

Fix $\lambda >0$ and give two different IFVs $\alpha =\langle \mu _{\alpha }, \nu _{\alpha }\rangle$ and $\beta = \langle \mu _{\beta }, \nu _{\beta }\rangle$ with $\mu _{\alpha }+\nu _{\alpha }\le \frac{\lambda }{2}$ and $\mu _{\beta } +\nu _{\beta }\le \frac{\lambda }{2}$. By differential mean value theorem, it can be verified that

(i) If $\mu _{\beta }\ge \mu _{\alpha }$ and $\nu _{\beta }\ge \nu _{\alpha }$, then there exist $\xi _1\in [\mu _{\alpha }, \mu _{\beta }]$ and $\eta _1\in [\nu _{\alpha }, \nu _{\beta }]$ such that $d_{_{\textrm{pd}}}^{(\lambda )}(\alpha , \beta )=\frac{2\mu _{\alpha }+2\nu _{\alpha }+\lambda }{(\mu _{\alpha }+\xi _1+\nu _{\alpha }+\eta _1+\lambda )^{2}}\cdot \frac{2+\lambda }{2}\cdot (\mu _{\beta }-\mu _{\alpha })+\frac{2\mu _{\alpha }+2\nu _{\alpha }+\lambda }{(\mu _{\alpha }+\xi _1+\nu _{\alpha }+\eta _1+\lambda )^{2}}\cdot \frac{2+\lambda }{2} \cdot (\nu _{\beta }-\nu _{\alpha })\ge \frac{\lambda }{(2\lambda )^2}\cdot \frac{2+\lambda }{2} \cdot ((\mu _{\beta }-\mu _{\alpha })+(\nu _{\beta }-\nu _{\alpha })) \ge \frac{1}{4\lambda }\cdot (|\mu _{\beta }-\mu _{\alpha }|+|\nu _{\beta }-\nu _{\alpha }|)$;
(ii) If $\mu _{\alpha }\ge \mu _{\beta }$ and $\nu _{\alpha }\ge \nu _{\beta }$, similarly to (i), it follows that there exist $\xi _2\in [\mu _{\beta }, \mu _{\alpha }]$ and $\eta _2\in [\nu _{\beta }, \nu _{\alpha }]$ such that $d_{_{\textrm{pd}}}^{(\lambda )}(\alpha , \beta )\ge \frac{1}{4\lambda } \cdot (|\mu _{\beta }-\mu _{\alpha }|+|\nu _{\beta }-\nu _{\alpha }|)$.
(iii) If $\mu _{\beta }\ge \mu _{\alpha }$ and $\nu _{\beta }\le \nu _{\alpha }$, then there exist $\xi _3\in [\mu _{\alpha }, \mu _{\beta }]$ and $\eta _3\in [\nu _{\beta }, \nu _{\alpha }]$ such that $d_{_{\textrm{pd}}}^{(\lambda )}(\alpha , \beta )=\frac{2\mu _{\alpha }+2\eta _3+\lambda }{(\mu _{\alpha }+\xi _3+\nu _{\alpha }+\eta _3+\lambda )^{2}}\cdot \frac{2+\lambda }{2} \cdot (\mu _{\beta }-\mu _{\alpha })+\frac{-2\xi _3-2\nu _{\alpha }-\lambda }{(\mu _{\alpha }+\xi _3+\nu _{\alpha }+\eta _3+\lambda )^{2}}\cdot \frac{2+\lambda }{2} \cdot (\nu _{\beta }-\nu _{\alpha })=\frac{2\mu _{\alpha }+2\eta _3+\lambda }{(\mu _{\alpha }+\xi _3+\nu _{\alpha }+\eta _3+\lambda )^{2}}\cdot \frac{2+\lambda }{2} \cdot (\mu _{\beta }-\mu _{\alpha })+\frac{2\xi _3+2\nu _{\alpha }+\lambda }{(\mu _{\alpha }+\xi _3+\nu _{\alpha }+\eta _3+\lambda )^{2}}\cdot \frac{2+\lambda }{2} \cdot (\nu _{\alpha }-\nu _{\beta })\ge \frac{\lambda }{(2\lambda )^2}\cdot \frac{2+\lambda }{2} \cdot ((\mu _{\beta }-\mu _{\alpha })+(\nu _{\alpha }-\nu _{\beta })) \ge \frac{1}{4\lambda }\cdot (|\mu _{\beta }-\mu _{\alpha }|+|\nu _{\beta }-\nu _{\alpha }|)$;
(iv) If $\mu _{\beta }\le \mu _{\alpha }$ and $\nu _{\beta }\ge \nu _{\alpha }$, similarly to (iii), it follows that there exist $\xi _4\in [\mu _{\beta }, \mu _{\alpha }]$ and $\eta _4\in [\nu _{\alpha }, \nu _{\beta }]$ such that $d_{_{\textrm{pd}}}^{(\lambda )}(\alpha , \beta ) \ge \frac{1}{4\lambda }\cdot (|\mu _{\beta }-\mu _{\alpha }|+|\nu _{\beta }-\nu _{\alpha }|)$.

According to the above theoretical analysis and also the presentation in Fig. 1, we can find that, when the parameter $\lambda$ is sufficiently small, the distance $d_{_{\textrm{pd}}}^{(\lambda )}$ can reach very large numbers and is sensitive to small perturbations, even if the degrees of membership and nonmembership are very small. Thus, the smaller the parameter $\lambda$ is, the stronger the sensitivity is. Therefore, the proposed parametric distance $d_{_{\textrm{pd}}}^{(\lambda )}$ can better distinguish IFVs with small degrees of membership and nonmembership. And so, throughout this paper, the values of the parameter $\lambda$ are chosen smaller. Meanwhile, according to Eq. (4), it is clear that the value of $d_{_{\textrm{pd}}}^{(\lambda )}$ will be sufficiently close to $\frac{1}{2} (|\mu _{\alpha }-\mu _{\beta }|+|\nu _{\alpha }-\nu _{\beta }|)$, when the parameter $\lambda$ is sufficiently higher. In this case, the distance measure $d_{_{\textrm{pd}}}^{(\lambda )}$ cannot distinguish different IFSs with high hesitancy, when the parameter $\lambda$ is sufficiently higher. In this sense, the values of the parameter $\lambda$ will not be chosen too high, but better with smaller values.

### The proposed IFDisM, IFSisM and IFEM for IFSs

Following the newly defined function $\textbf{d}_{_{\textrm{pd}}}^{(\lambda )}$ on $\Theta$ in “[Sec Sec8]”, we now propose the new IFDisM (distance), IFSisM (similarity) and IFEM (entropy) for IFSs as follows. Let $\Xi =\{\vartheta _1, \vartheta _2, \ldots , \vartheta _{\ell }\}$ and $\lambda >0$. Define the function $\textbf{d}_{_{\textrm{New}}}^{(\lambda )}: \textrm{IFS}(\Xi )\times \textrm{IFS}(\Xi ) \longrightarrow \mathbb {R}^{+}$ for $I_1=\{\langle \mu _{I_1}(\vartheta _i), \nu _{I_1}(\vartheta _i) \rangle \mid \vartheta _i\in \Xi \}$ and $I_2=\{\langle \mu _{I_2}(\vartheta _i), \nu _{I_2}(\vartheta _i) \rangle \mid \vartheta _i\in \Xi \}\in \textrm{IFS}(\Xi )$,

9 $$\begin{aligned} \textbf{d}_{_{\textrm{New}}}^{(\lambda )}(I_1, I_2)= \sum _{i=1}^{\ell }\omega _{i} \cdot d_{_{\textrm{New}}}^{(\lambda )} (\langle \mu _{I_1}(\vartheta _i), \nu _{I_1}(\vartheta _i) \rangle , \langle \mu _{I_2}(\vartheta _i), \nu _{I_2}(\vartheta _i) \rangle ), \end{aligned}$$

where $\omega =(\omega _1, \omega _2, \ldots , \omega _n)^{\top }$ is the weight vector of $\vartheta _{i}$ ($i=1, 2, \ldots , \ell$) with $\omega _i\in (0, 1]$ and $\sum _{i=1}^{\ell }\omega _i=1$.

Based on the defined IFDisM $\textbf{d}_{_{\textrm{New}}}^{(\lambda )}(I_1, I_2)$ for IFSs, we can define a new similarity measure $\textbf{S}_{_{\textrm{New}}}^{(\lambda )}(I_1, I_2)$ for IFSs as follows: for $I_1=\{\langle \mu _{I_1}(\vartheta _i), \nu _{I_1}(\vartheta _i) \rangle \mid \vartheta _i\in \Xi \}$ and $I_2=\{\langle \mu _{I_2}(\vartheta _i), \nu _{I_2}(\vartheta _i) \rangle \mid \vartheta _i\in \Xi \}\in \textrm{IFS}(\Xi )$,

10 $$\begin{aligned} \textbf{S}_{_{\textrm{New}}}^{(\lambda )}(I_1, I_2)=1-\textbf{d}_{_{\textrm{New}}}^{(\lambda )}(I_1, I_2) \end{aligned}$$

Similarly, a new entropy measure for IFSs can be defined according to the defined IFDisM $\textbf{d}_{_{\textrm{New}}}^{(\lambda )}(I_1, I_2)$ as follows:

11 $$\begin{aligned} \begin{aligned} E: \textrm{IFS}(\Xi )&\longrightarrow [0, 1], \\ I&\longmapsto 1-\textbf{d}_{_{\textrm{New}}}^{(\lambda )} (I, I^{\complement }), \end{aligned} \end{aligned}$$

According to Theorems 3.1 and 3.2, we can directly obtain the following theorems.

#### Theorem 3.3

1. *The function* $\textbf{d}_{_{\textrm{New}}}^{(\lambda )}$ *defined by Eq*. (9) *is a strict IFDisM on* $\textrm{IFS}(\Xi )$.
2. *The function* $\textbf{S}_{_{\textrm{New}}}^{(\lambda )}(I_1, I_2)$ *defined by Eq*. (10) *is a strict IFSimM on* $\textrm{IFS}(\Xi )$.

#### Theorem 3.4

*Let* $\lambda >0$. *The measure* *E* *defined by Eq*. (11) *is an entropy measure on* $\textrm{IFS}(\Xi )$.

### Comparative analysis with Mahanta and Panda’s distance measure

This subsection illustrates that our proposed distance measure can completely overcome Mahanta and Panda’s drawbacks mentioned in “The drawbacks of distance measure of Mahanta and Panda^38^”.

#### *Example* 3.3

(Continuation of Example 3.1) Take the IFSs $I_1$ on $\Xi =\{\vartheta \}$ as given in Example 3.1. For any $I_2=\left\{ \frac{\langle \mu , \nu \rangle }{\vartheta }\right\} \in \textrm{IFS}(\Xi )$ with $I_2\ne I_1$, by direct calculation and Eq. (9), we have

$$\begin{aligned} \textbf{d}_{_{\textrm{New}}}^{(\lambda )}(I_1, I_2)= \frac{|\mu -0|+|\nu -0|}{\mu +0+\nu +0+\lambda }\cdot \frac{2+\lambda }{2} =\frac{\mu +\nu }{\mu +\nu +\lambda }\cdot \frac{2+\lambda }{2}. \end{aligned}$$

**Figure 2:**
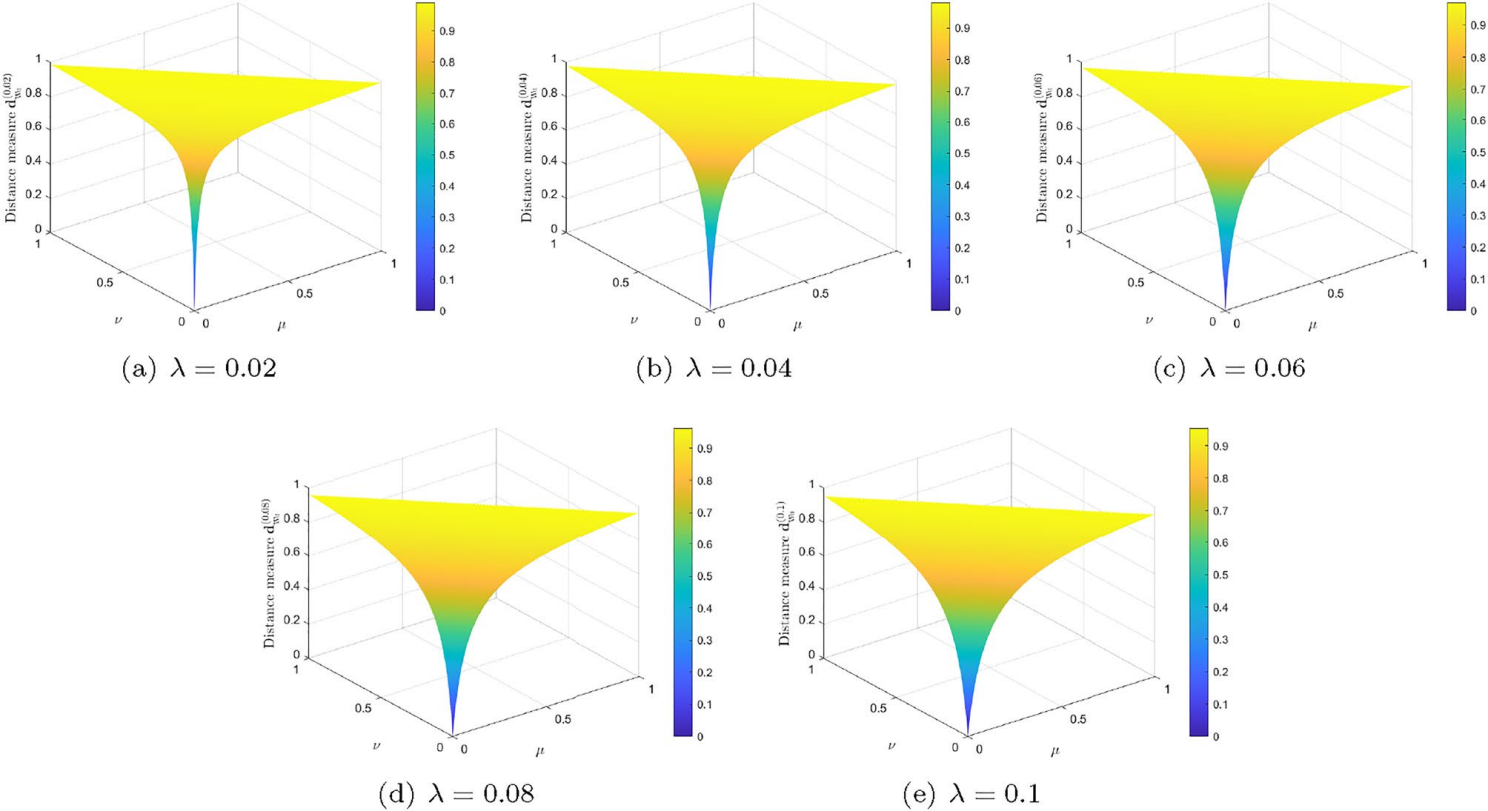
Distance measure $\textbf{d}_{_{\textrm{New}}}^{(\lambda )}(I_1, I_2)$ in Example 3.3 for different values of $\lambda$.

By varying IFS $I_2$ within $\textrm{IFS}(\Xi )$, Fig. 2 shows the changing trend of distances between $I_1$ and $I_2$ by using our proposed formula (9) for $\lambda =0.02, 0.04, 0.06, 0.08, 0.1$. Observing from Example 3.1, Proposition 3.4, and Fig. 2, it is revealed that the distance $\textbf{d}_{_{\textrm{New}}}^{(\lambda )}(I_1, I_2)$ between $I_1$ and $I_2$ is always less than 1, and changed with the change of $I_{2}$, which are reasonable, and significantly better than the result obtained by Mahanta and Panda’s distance measure in Example 3.1.

#### *Example* 3.4

**Figure 3:**
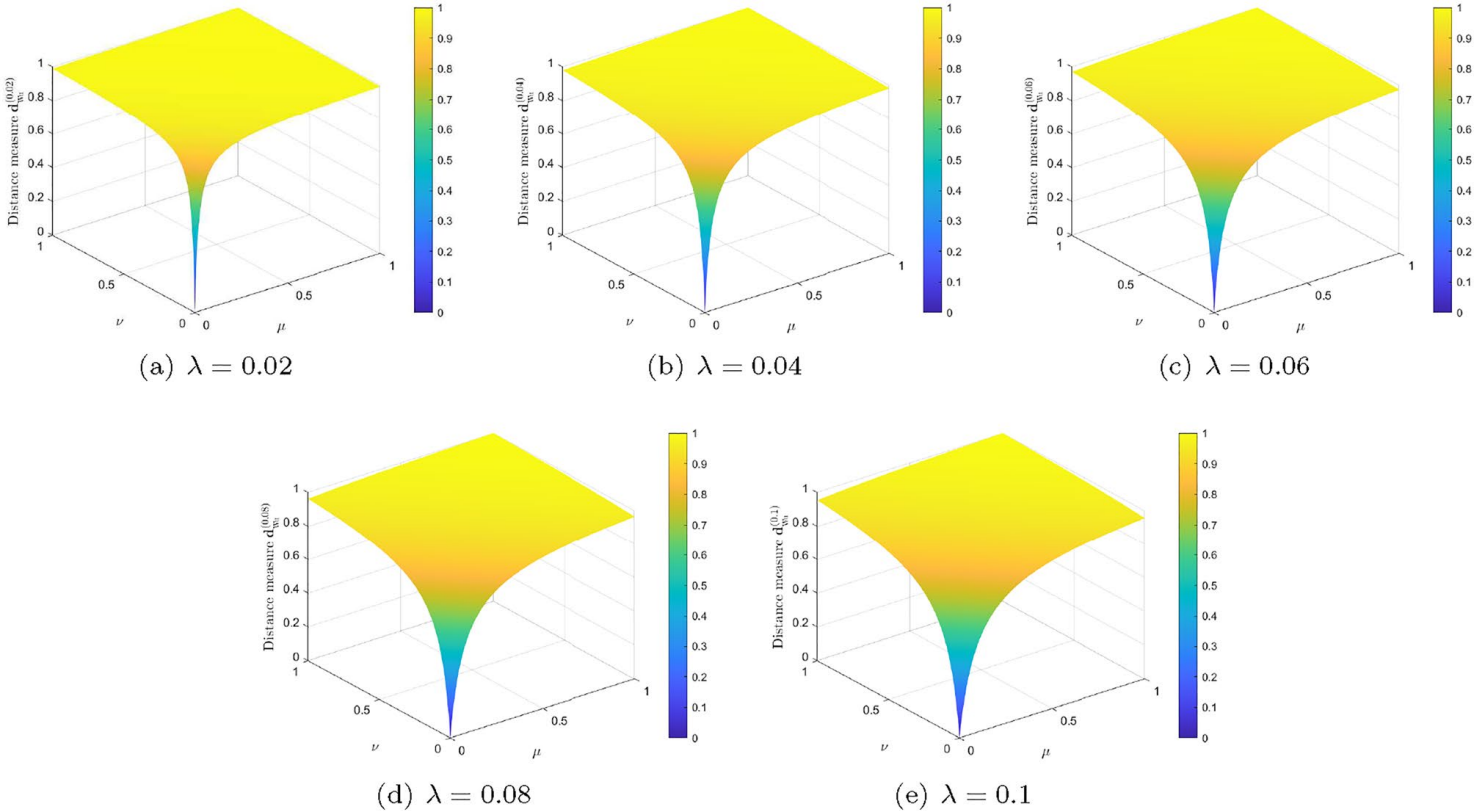
Distance measure $\textbf{d}_{_{\textrm{New}}}^{(\lambda )}(I_1^{\prime }, I_2^{\prime })$ in Example 3.4 for different values of $\lambda$.

(Continuation of Example 3.2) Take the IFSs $I_1^{\prime }$ and $I_{2}^{\prime }$ on $\Xi =\{\vartheta \}$ as given in Example 3.2. By direct calculation and Eq. (9), we have that, for $0<\mu , \nu \le 1$, $\textbf{d}_{_{\textrm{New}}}^{(\lambda )}(I_1^{\prime }, I_2^{\prime })= \frac{|\mu -0|+|0-\nu |}{\mu +0+0+\nu +\lambda }\cdot \frac{2+\lambda }{2} =\frac{\mu +\nu }{\mu +\nu +\lambda }\cdot \frac{2+\lambda }{2}.$ By varying $\mu$ and $\nu$ from 0 to 1, Fig. 3 shows the changing trend of distances between $I_1^{\prime }$ and $I_2^{\prime }$ by using our proposed formula (9) for $\lambda =0.02, 0.04, 0.06, 0.08, 0.1$. Observing from Example 3.2, Proposition 3.4, and Fig. 3, it is revealed that the distance $\textbf{d}_{_{\textrm{New}}}^{(\lambda )}(I_1^{\prime }, I_2^{\prime })$ between $I_1^{\prime }$ and $I_2^{\prime }$ is always less than 1 except for $\mu =\nu =1$, and changes with the changed of $I_{1}^{\prime }$ and $I_{2}^{\prime }$, which are reasonable, and significantly better than the result obtained by Mahanta and Panda’s distance measure in Example 3.2.

## Applications

### A pattern classification problem

#### *Example* 4.1

(^32^Application 2, ^14^Example 4.3) Consider a pattern classification problem with three classes and three attributes ${\mathfrak {A}}=\{x_1, x_2, x_3\}$, described by three patterns ${\mathfrak {P}}=\{P_1, P_2, P_3\}$ and a test sample $S_1$ expressed by the IFSs listed in Table 1.

**Figure 4:**
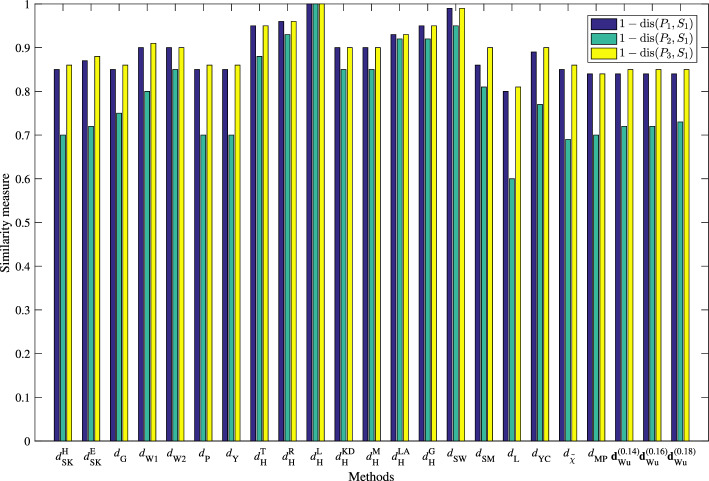
Comparison results of different similarity measures in Example 4.1.

By taking the weight vector $\omega$ of three attributes as $\omega =(\frac{1}{3}, \frac{1}{3}, \frac{1}{3})^{\top }$, based on the principle of the maximum degree of SimMs, the pattern classification results obtained by using different distance measures are listed in Table 2 and Fig. 4. Observing from Table 2 and Fig. 4, we know that the test sample $S_{1}$ is classified to the pattern $P_3$ by our proposed DisM with $\lambda =0.14, 0.16, 0.18$, which is consistent with the results obtained by the DisMs $d_{_{\textrm{SK}}}^{\textrm{E}}$, $d_{_{\textrm{G}}}$, $d_{_{\textrm{W1}}}$, $d_{_{\textrm{W2}}}$, $d_{_{\textrm{P}}}$, $d_{_{\textrm{Y}}}$, $d_{_{\textrm{SW}}}$, $d_{_{\textrm{SM}}}$, $d_{_{\textrm{L}}}$, $d_{_{\textrm{YC}}}$, and $d_{\widetilde{\chi }}$; However, the methods using DisMs $d_{_{\textrm{W2}}}$, $d_{_{\textrm{H}}}^{\textrm{T}}$, $d_{_{\textrm{H}}}^{\textrm{R}}$, $d_{_{\textrm{H}}}^{\textrm{L}}$, $d_{_{\textrm{H}}}^{\textrm{KD}}$, $d_{_{\textrm{H}}}^{\textrm{M}}$, $d_{_{\textrm{H}}}^{\textrm{LA}}$, $d_{_{\textrm{H}}}^{\textrm{G}}$, $d_{_{\textrm{SW}}}$, and $d_{_{\textrm{MP}}}$ cannot determine to which pattern the test sample $S_1$ belongs. We mention that the calculations for $d_{_{\textrm{MP}}}$ by Mahanta and Panda^38^ have $1-d_{_{\textrm{MP}}}(P_1, S_1)=0.8354$ and $1-d_{_{\textrm{MP}}}(P_3, S_1)=0.8383$. This means that $1-d_{_{\textrm{MP}}}(P_3, S_1)>1-d_{_{\textrm{MP}}}(P_1, S_1)$, and so it is able to distinguish between the patterns, but only a little. However, if we retain 2 digits after the decimal point, we have $1-d_{_{\textrm{MP}}}(P_3, S_1)=0.84=1-d_{_{\textrm{MP}}}(P_1, S_1)$, and so $d_{_{\textrm{MP}}}$ by Mahanta and Panda^38^ can not distinguish between the patterns.

**Table 1:** Pattern classification in Example 4.1.

	Attribute					
	$x_{1}$		$x_{2}$		$x_{3}$	
	$\mu _{P}(x_1)$	$\nu _{P}(x_1)$	$\mu _{P}(x_2)$	$\nu _{P}(x_2)$	$\mu _{P}(x_3)$	$\nu _{P}(x_3)$
$$\text {Pattern}$$						
$P_1$	0.15	0.25	0.25	0.35	0.35	0.45
$P_2$	0.05	0.15	0.15	0.25	0.25	0.35
$P_3$	0.16	0.26	0.26	0.36	0.36	0.46
$$\text {Sample}$$						
$S_1$	0.30	0.20	0.40	0.30	0.50	0.40

**Table 2:** Pattern recognition results by different similarity measures in Example 4.1.

Method	Similarity measure			Classification
	$1-\text {dis}(P_1, S_1)$	$1-\text {dis}(P_2, S_1)$	$1-\text {dis}(P_3, S_1)$	
$d_{_{\textrm{SK}}}^{\textrm{H}}$ in^33^	0.85	0.70	0.86	$P_{3}$
$d_{_{\textrm{SK}}}^{\textrm{E}}$ in^33^	0.87	0.72	0.88	$P_{3}$
$d_{_{\textrm{G}}}$ in^29^	0.85	0.75	0.86	$P_{3}$
$d_{_{\textrm{W1}}}$ in^23^	0.90	0.80	0.91	$P_{3}$
$d_{_{\textrm{W2}}}$ in^23^	0.90	0.85	0.90	✕
$d_{_{\textrm{P}}}$ in^40^	0.85	0.70	0.86	$P_{3}$
$d_{_{\textrm{Y}}}$ in^34^	0.85	0.70	0.86	$P_{3}$
$d_{_{\textrm{H}}}^{\textrm{T}}$ in^41^	0.95	0.88	0.95	✕
$d_{_{\textrm{H}}}^{\textrm{R}}$ in^41^	0.96	0.93	0.96	✕
$d_{_{\textrm{H}}}^{\textrm{L}}$ in^41^	$1-3.70\times 10^{-17}$	$1-3.70\times 10^{-17}$	$1-3.70\times 10^{-17}$	✕
$d_{_{\textrm{H}}}^{\textrm{KD}}$ in^41^	0.90	0.85	0.90	✕
$d_{_{\textrm{H}}}^{\textrm{M}}$ in^41^	0.90	0.85	0.90	✕
$d_{_{\textrm{H}}}^{\textrm{LA}}$ in^41^	0.93	0.92	0.93	✕
$d_{_{\textrm{H}}}^{\textrm{G}}$ in^41^	0.95	0.92	0.95	✕
$d_{_{\textrm{SW}}}$ in^36^	0.99	0.95	0.99	✕
$d_{_{\textrm{SM}}}$ in^35^	0.86	0.81	0.90	$P_{3}$
$d_{_{\textrm{L}}}$ in^14^	0.80	0.60	0.81	$P_{3}$
$d_{_{\textrm{YC}}}$ in^42^	0.89	0.77	0.90	$P_{3}$
$d_{\widetilde{\chi }}$ in^32^	0.85	0.69	0.86	$P_{3}$
$d_{_{\textrm{MP}}}$ in^38^	0.84	0.70	0.84	✕
$\textbf{d}_{_{\textrm{New}}}^{(0.14)}$	0.84	0.72	0.85	$P_{3}$
$\textbf{d}_{_{\textrm{New}}}^{(0.16)}$	0.84	0.72	0.85	$P_{3}$
$\textbf{d}_{_{\textrm{New}}}^{(0.18)}$	0.84	0.73	0.85	$P_{3}$

✕ denotes that it cannot be determined.

The details for distance measures in Table 2 can be found in^32^, Section III.

### A TOPSIS method based on our proposed strict IFDisM and IFSimM

Suppose that there are *n* alternatives ${A}_{i}$ ($i=1, 2, \ldots , n$) evaluated with respect to *m* attributes ${\mathfrak {A}}_j$ ($j=1, 2, \ldots , m$). The sets of the alternatives and attributes are denoted by $A=\{A_1, A_2, \ldots , A_n\}$ and ${\mathfrak {A}}=\{{\mathfrak {A}}_1, {\mathfrak {A}}_2, \ldots , {\mathfrak {A}}_m\}$, respectively. The rating (or evaluation) of each alternative $A_i\in A$ ($i=1, 2, \ldots , n$) on each attribute $o_j$ ($j=1, 2, \ldots , m$) is expressed with an IFS $r_{ij}= \left\{ \frac{\langle \mu _{ij}, \nu _{ij}\rangle }{(A_i, {\mathfrak {A}}_j)}\right\}$, denoted by $r_{ij}=\langle \mu _{ij}, \nu _{ij}\rangle$ for short, where $\mu _{ij}\in [0, 1]$ and $\nu _{ij}\in [0, 1]$ are respectively the satisfaction (or membership) degree and dissatisfaction (or non-membership) degree of the alternative $A_{i}\in A$ on the attribute $o_{j}$ satisfying the condition $0\le \mu _{ij}+\nu _{ij}\le 1$. A multi-attribute decision-making (MADM) problem with IFSs is expressed in matrix form shown in Table 3.

**Table 3:** IF decision matrix $R=(r_{ij})_{n\times m}$.

	${\mathfrak {A}}_{1}$	${\mathfrak {A}}_{2}$	$\ldots$	${\mathfrak {A}}_{m}$
$A_{1}$	$\langle \mu _{11}, \nu _{11}\rangle$	$\langle \mu _{12}, \nu _{12}\rangle$	$\ldots$	$\langle \mu _{1m}, \nu _{1m}\rangle$
$A_{2}$	$\langle \mu _{21}, \nu _{21}\rangle$	$\langle \mu _{22}, \nu _{22}\rangle$	$\ldots$	$\langle \mu _{2m}, \nu _{2m}\rangle$
$\vdots$	$\vdots$	$\vdots$	$\ddots$	$\vdots$
$A_{n}$	$\langle \mu _{n1}, \nu _{n1}\rangle$	$\langle \mu _{n2}, \nu _{n2}\rangle$	$\ldots$	$\langle \mu _{nm}, \nu _{nm}\rangle$

For the MADM problem with IFSs, by using our proposed IFDisM $\textbf{d}_{_{\textrm{New}}}^{(\lambda )}$ of Eq. (9), we construct a new IF TOPSIS method as follows:

Step 1: (Construct the decision matrix) Supposing that the decision-maker gave the rating (or evaluation) of each alternative $A_i\in A$ ($i=1, 2, \ldots , n$) on each attribute ${\mathfrak {A}}_j$ ($j=1, 2, \ldots , m$) in the form of IFNs $r_{ij}=\langle \mu _{ij}, \eta _{ij}\rangle$, construct an IF decision matrix $R=(r_{ij})_{m\times n}$ as shown in Table 3.

Step 2: (Normalize the decision matrix) Transform the IF decision matrix $R=(r_{ij})_{m\times n}$ to the normalized IF decision matrix $\overline{R}=(\bar{r}_{ij})_{m\times n}=(\langle \bar{\mu }_{ij}, \bar{\nu }_{ij}\rangle )_{m\times n}$ as follows:

$$\begin{aligned} \bar{r}_{ij}= {\left\{ \begin{array}{ll} r_{ij}, &{} \text {for benefit attribute } {\mathfrak {A}}_{j}, \\ r_{ij}^{\complement }, &{} \text {for cost attribute } {\mathfrak {A}}_{j}, \end{array}\right. } \end{aligned}$$

where $r_{ij}^{\complement }$ is the complement of $\gamma _{ij}$.

Step 3: (Determine the positive and negative ideal-points) Determine the IF positive ideal-point ${\mathfrak {I}}^{+}=(\langle \mu ^{+}_{1}, \nu ^{+}_{1}\rangle , \langle \mu ^{+}_{2}, \nu ^{+}_{2}\rangle ,$ $\ldots , \langle \mu ^{+}_{m}, \nu ^{+}_{m}\rangle )^{\top }$ and IF negative ideal-point ${\mathfrak {I}}^{-}=(\langle \mu ^{-}_{1}, \nu ^{-}_{1}\rangle , \langle \mu ^{-}_{2}, \nu ^{-}_{2}\rangle , \ldots , \langle \mu ^{-}_{m}, \nu ^{-}_{m}\rangle )^{\top }$ as follows:

$$\begin{aligned}&\mu ^{+}_{j}=\max _{1\le i\le n}\{\bar{\mu }_{ij}\}, \quad \nu ^{+}_{j}=\min _{1\le i\le n}\{\bar{\nu }_{ij}\},\\&\mu ^{-}_{j}=\min _{1\le i\le n}\{\bar{\mu }_{ij}\}, \quad \nu ^{-}_{j}=\max _{1\le i\le n}\{\bar{\nu }_{ij}\}. \end{aligned}$$

Step 4: (Compute the weighted similarity measures) Compute the weighted similarity measures between the alternatives $A_i$ ($i=1,2, \ldots ,n$) and the IF positive ideal-point ${\mathfrak {I}}^+$, and between the alternatives $A_i$ ($i=1,2, \ldots ,n$) and the IF negative ideal-point ${\mathfrak {I}}^{-}$, by using the following formulas:

12 $$\begin{aligned} \textbf{S}(A_i, {\mathfrak {I}}^{+})=1-\sum _{j=1}^{m}\omega _{j}\cdot d_{_{\textrm{New}}}^{(\lambda )}(\langle \bar{\mu }_{ij}, \bar{\nu }_{ij}\rangle , \langle \mu ^{+}_{j}, \nu ^{+}_{j}\rangle ), \end{aligned}$$

and

13 $$\begin{aligned} \textbf{S}(A_i, {\mathfrak {I}}^{-})=1-\sum _{j=1}^{m}\omega _{j}\cdot d_{_{\textrm{New}}}^{(\lambda )}(\langle \bar{\mu }_{ij}, \bar{\nu }_{ij}\rangle , \langle \mu ^{-}_{j}, \nu ^{-}_{j}\rangle ). \end{aligned}$$

Step 5: (Compute the relative similarity degrees) Calculate the relative similarity degrees ${\mathcal {C}}_{i}$ of the alternatives $A_i$ ($i=1,2, \ldots ,n$) to the IF positive ideal-point ${\mathfrak {I}}^+$ by using the following formula:

14 $$\begin{aligned} {\mathcal {C}}_{i}=\frac{\textbf{S}(A_i, {\mathfrak {I}}^{+})}{\textbf{S}(A_i, {\mathfrak {I}}^{+})+\textbf{S}(A_i, {\mathfrak {I}}^{-})}. \end{aligned}$$

Step 6: (Rank the alternative) Rank the alternatives $A_i$ ($i=1,2, \ldots ,n$) according to the nonincreasing order of the relative closeness degrees ${\mathcal {C}}_{i}$ and select the most desirable alternative.

### Comparative and sensitivity analysis

We next make a comparative and sensitivity analysis of our proposed method with the method proposed by Mahanta and Panda^38^.

#### *Example* 4.2

(^38^Example 4.3) After the outbreak of COVID-19 disease, the demand for masks has increased rapidly. There are six common types of masks on the market as follows: ${\mathscr {M}}_1$—disposable medical masks, ${\mathscr {M}}_2$—medical-surgical masks, ${\mathscr {M}}_3$—particulate respirators (N95), ${\mathscr {M}}_4$—ordinary nonmedical masks, ${\mathscr {M}}_5$—medical protective masks, and ${\mathscr {M}}_6$—gas masks. A citizen wants to buy a suitable mask from the above six types of masks by considering the following four attributes: ${\mathfrak {A}}_1$—leakage rate, ${\mathfrak {A}}_2$—recyclability, ${\mathfrak {A}}_3$—quality of raw material, ${\mathfrak {A}}_4$—filtration capability.

Step 1: (Construct the decision matrix) Through the market survey, the evaluations of each type of mask ${\mathscr {M}}_i$ ($i=1, 2, 3, 4, 5, 6$) on each attribute ${\mathfrak {A}}_j$ ($j=1, 2, 3, 4$) in the form of IFNs are summarized in Table 4.

**Table 4:** IFN evaluation of different types of masks.

Mask type	${\mathfrak {A}}_{1}$	${\mathfrak {A}}_{2}$	${\mathfrak {A}}_{3}$	${\mathfrak {A}}_{4}$
${\mathscr {M}}_{1}$	$\langle 0.5329, 0.0841\rangle$	$\langle 0.6400,0.0144\rangle$	$\langle 0.0784, 0.1936 \rangle$	$\langle 0.0784, 0.4624\rangle$
${\mathscr {M}}_{2}$	$\langle 0.0841, 0.3721\rangle$	$\langle 0.2916,0.3969\rangle$	$\langle 0.0729,0.4624\rangle$	$\langle 0.0900, 0.3960\rangle$
${\mathscr {M}}_{3}$	$\langle 0.2916, 0.2401\rangle$	$\langle 0.1936,0.3136\rangle$	$\langle 0.3721,0.2916\rangle$	$\langle 0.5329,0.1764\rangle$
${\mathscr {M}}_{4}$	$\langle 0.1521, 0.4096\rangle$	$\langle 0.1156, 0.1849\rangle$	$\langle 0.2025, 0.0961\rangle$	$\langle 0.0529,0.3721\rangle$
${\mathscr {M}}_{5}$	$\langle 0.2809,0.0841\rangle$	$\langle 0.2025, 0.4356\rangle$	$\langle 0.5329, 0.1936\rangle$	$\langle 0.3600, 0.3969\rangle$
${\mathscr {M}}_{6}$	$\langle 0.0100, 0.0625\rangle$	$\langle 0.1024, 0.0729\rangle$	$\langle 0.1849, 0.1369\rangle$	$\langle 0.3600,0.3600\rangle$

Step 2: (Normalize the decision matrix) Because ${\mathfrak {A}}_1$ is a cost attribute and ${\mathfrak {A}}_2$–${\mathfrak {A}}_4$ are the benefit attributes, the normalized IF decision matrix is formed as shown in Table 5.

**Table 5:** Normalized IFN evaluation of different types of masks.

Mask type	${\mathfrak {A}}_{1}$	${\mathfrak {A}}_{2}$	${\mathfrak {A}}_{3}$	${\mathfrak {A}}_{4}$
${\mathscr {M}}_{1}$	$\langle 0.0841, 0.5329\rangle$	$\langle 0.6400, 0.0144\rangle$	$\langle 0.0784,0.1936\rangle$	$\langle 0.0784, 0.4624 \rangle$
${\mathscr {M}}_{2}$	$\langle 0.3721, 0.0841\rangle$	$\langle 0.2916,0.3969\rangle$	$\langle 0.0729,0.4624\rangle$	$\langle 0.0900, 0.3960\rangle$
${\mathscr {M}}_{3}$	$\langle 0.2401, 0.2916 \rangle$	$\langle 0.1936,0.3136\rangle$	$\langle 0.3721,0.2916\rangle$	$\langle 0.5329,0.1764\rangle$
${\mathscr {M}}_{4}$	$\langle 0.4096, 0.1521\rangle$	$\langle 0.1156,0.1849\rangle$	$\langle 0.2025,0.0961\rangle$	$\langle 0.0529,0.3721\rangle$
${\mathscr {M}}_{5}$	$\langle 0.0841, 0.2809 \rangle$	$\langle 0.2025,0.4356\rangle$	$\langle 0.5329,0.1936\rangle$	$\langle 0.3600, 0.3969 \rangle$
${\mathscr {M}}_{6}$	$\langle 0.0625, 0.0100 \rangle$	$\langle 0.1024, 0.0729\rangle$	$\langle 0.1849, 0.1369\rangle$	$\langle 0.3600, 0.3600 \rangle$

Step 3: (Determine the positive and negative ideal-points) The IF positive ideal-point is

$$\begin{aligned} {\mathfrak {I}}^{+}=\{\langle 0.4096, 0.0100 \rangle , \langle 0.6400, 0.0144 \rangle , \langle 0.5329, 0.0961 \rangle , \langle 0.5329, 0.1764 \rangle \}, \end{aligned}$$

and the IF negative ideal-point is

$$\begin{aligned} {\mathfrak {I}}^{-}=\{\langle 0.0625, 0.5329 \rangle , \langle 0.1024, 0.4356 \rangle , \langle 0.0729, 0.4624 \rangle , \langle 0.0529, 0.4624 \rangle \}. \end{aligned}$$

Steps 4 and 5: (Compute the relative similarity degrees) Take the weight vector $\omega =(0.25, 0.25, 0.25, 0.25)^{\top }$. For $\lambda =0.02$, 0.04, 0.06, 0.08, 0.1, calculate the relative similarity degrees ${\mathcal {C}}_{i}$ of the alternatives ${\mathscr {M}}_i$ ($i=1, 2, 3, 4, 5, 6$) to the IF positive ideal-point ${\mathfrak {I}}^+$ by Eqs. (12), (13), and (14). The results are presented in Table 6.

**Table 6:** Relative similarity degrees ${\mathcal {C}}_{i}$ with $\omega =(0.25, 0.25, 0.25, 0.25)^{\top }$.

Relative similarity	${\mathcal {C}}_{1}$	${\mathcal {C}}_{2}$	${\mathcal {C}}_{3}$	${\mathcal {C}}_{4}$	${\mathcal {C}}_{5}$	${\mathcal {C}}_{6}$	Ranking
$\lambda =0.02$	0.4139	0.4093	0.5200	0.4792	0.4486	0.5071	${\mathscr {M}}_3\succ {\mathscr {M}}_6\succ {\mathscr {M}}_4\succ {\mathscr {M}}_5 \succ {\mathscr {M}}_1\succ {\mathscr {M}}_2$
$\lambda =0.04$	0.4152	0.4101	0.5199	0.4794	0.4495	0.5073	${\mathscr {M}}_3\succ {\mathscr {M}}_6\succ {\mathscr {M}}_4\succ {\mathscr {M}}_5 \succ {\mathscr {M}}_1\succ {\mathscr {M}}_2$
$\lambda =0.06$	0.4164	0.4108	0.5198	0.4795	0.4504	0.5075	${\mathscr {M}}_3\succ {\mathscr {M}}_6\succ {\mathscr {M}}_4\succ {\mathscr {M}}_5 \succ {\mathscr {M}}_1\succ {\mathscr {M}}_2$
$\lambda =0.08$	0.4175	0.4115	0.5197	0.4797	0.4513	0.5076	${\mathscr {M}}_3\succ {\mathscr {M}}_6\succ {\mathscr {M}}_4\succ {\mathscr {M}}_5 \succ {\mathscr {M}}_1\succ {\mathscr {M}}_2$
$\lambda =0.1$	0.4186	0.4122	0.5197	0.4798	0.4521	0.5077	${\mathscr {M}}_3\succ {\mathscr {M}}_6\succ {\mathscr {M}}_4\succ {\mathscr {M}}_5 \succ {\mathscr {M}}_1\succ {\mathscr {M}}_2$

Step 6: (Rank the alternative) For any $\lambda \in \{0.02, 0.04, 0.06, 0.08, 0.1\}$, because it always holds ${\mathcal {C}}_3> {\mathcal {C}}_6> {\mathcal {C}}_4> {\mathcal {C}}_5> {\mathcal {C}}_1> {\mathcal {C}}_2$, the ranking of these types of masks ${\mathscr {M}}_i$ ($i=1, 2, 3, 4, 5, 6$) is:

$$\begin{aligned} {\mathscr {M}}_3\succ {\mathscr {M}}_6\succ {\mathscr {M}}_4\succ {\mathscr {M}}_5 \succ {\mathscr {M}}_1\succ {\mathscr {M}}_2. \end{aligned}$$

Therefore, the most desirable mask type is ${\mathscr {M}}_3$—particulate respirators (N95).

Mahanta and Panda^38^, Example 4.3 showed that the most desirable mask type is ${\mathscr {M}}_1$—disposable medical masks, which is different from our result. The main reason for this is lack of normalization step (Step 2) in Mahanta and Panda’s TOPSIS method^38^. This may yield counter-intuitive results, because the smaller the score for cost attribute is, the better the attribute on this attribute is. To illustrate the effectiveness of the proposed TOPSIS method, we give a comparison of the preference orders of the alternatives in Example 4.2 for different TOPSIS methods as follows.

**Table 7:** A comparison of the ranking for the alternatives in Example 4.2 for different TOPSIS methods.

Relative similarity	${\mathcal {C}}_{1}$	${\mathcal {C}}_{2}$	${\mathcal {C}}_{3}$	${\mathcal {C}}_{4}$	${\mathcal {C}}_{5}$	${\mathcal {C}}_{6}$	Ranking
$\lambda =0.02$	0.4139	0.4093	0.5200	0.4792	0.4486	0.5071	${\mathscr {M}}_3\succ {\mathscr {M}}_6\succ {\mathscr {M}}_4\succ {\mathscr {M}}_5 \succ {\mathscr {M}}_1\succ {\mathscr {M}}_2$
$\lambda =0.04$	0.4152	0.4101	0.5199	0.4794	0.4495	0.5073	${\mathscr {M}}_3\succ {\mathscr {M}}_6\succ {\mathscr {M}}_4\succ {\mathscr {M}}_5 \succ {\mathscr {M}}_1\succ {\mathscr {M}}_2$
$\lambda =0.06$	0.4164	0.4108	0.5198	0.4795	0.4504	0.5075	${\mathscr {M}}_3\succ {\mathscr {M}}_6\succ {\mathscr {M}}_4\succ {\mathscr {M}}_5 \succ {\mathscr {M}}_1\succ {\mathscr {M}}_2$
$\lambda =0.08$	0.4175	0.4115	0.5197	0.4797	0.4513	0.5076	${\mathscr {M}}_3\succ {\mathscr {M}}_6\succ {\mathscr {M}}_4\succ {\mathscr {M}}_5 \succ {\mathscr {M}}_1\succ {\mathscr {M}}_2$
$\lambda =0.1$	0.4186	0.4122	0.5197	0.4798	0.4521	0.5077	${\mathscr {M}}_3\succ {\mathscr {M}}_6\succ {\mathscr {M}}_4\succ {\mathscr {M}}_5 \succ {\mathscr {M}}_1\succ {\mathscr {M}}_2$
TOPSIS method in^38^	0.5133	0.4636	0.5070	0.4797	0.5074	0.4981	${\mathscr {M}}_1\succ {\mathscr {M}}_5\succ {\mathscr {M}}_3\succ {\mathscr {M}}_6 \succ {\mathscr {M}}_4\succ {\mathscr {M}}_2$
TOPSIS method in^35^	− 0.1602	− 0.2023	0.0507	− 0.0514	− 0.0800	0.0420	${\mathscr {M}}_3\succ {\mathscr {M}}_6\succ {\mathscr {M}}_4\succ {\mathscr {M}}_5 \succ {\mathscr {M}}_1\succ {\mathscr {M}}_2$
TOPSIS method in^43^	0.4665	0.4505	0.5135	0.4907	0.4834	0.5009	${\mathscr {M}}_3\succ {\mathscr {M}}_6\succ {\mathscr {M}}_4\succ {\mathscr {M}}_5 \succ {\mathscr {M}}_1\succ {\mathscr {M}}_2$
TOPSIS method in^25^	0.5051	0.4175	0.5484	0.4895	0.4813	0.5018	${\mathscr {M}}_3\succ {\mathscr {M}}_1\succ {\mathscr {M}}_6\succ {\mathscr {M}}_4 \succ {\mathscr {M}}_5\succ {\mathscr {M}}_2$

From Table 7, which shows a comparison of the preference orders of the alternatives in Example 4.2 for different TOPSIS methods, we observe that although our ranking result is different from these obtained by the TOPSIS method in^25,35,43^, the most desirable mask type is always ${\mathscr {M}}_3$–particulate respirators (N95). Note that the scores of ${\mathscr {M}}_3$ on the attributes ${\mathfrak {A}}_2$, ${\mathfrak {A}}_3$, and ${\mathfrak {A}}_4$ (by Table 5) are much greater than that of ${\mathscr {M}}_1$. This gives a reason to support the conclusion that ${\mathscr {M}}_{3}$ is better than ${\mathscr {M}}_1$. Therefore, our method is more reasonable than that of Mahanta and Panda^38^.

**Figure 5:**
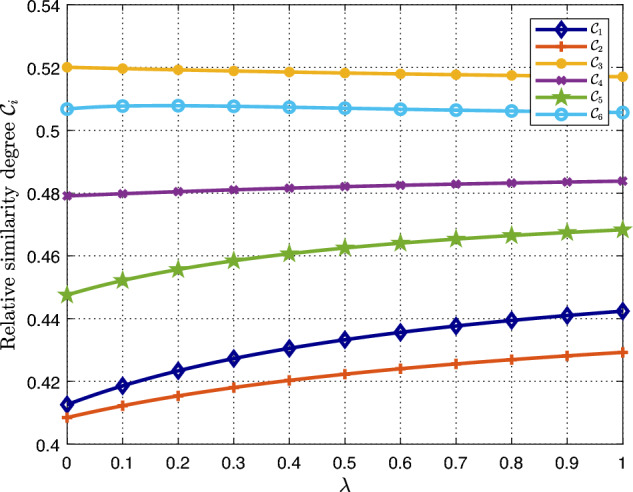
Relative similarity degrees for different values of $\lambda$ for $\omega =(0.25, 0.25, 0.25, 0.25)^{\top }$.

To study the changing tendency of the relative similarity degrees and the rankings for ${\mathscr {M}}_1$, ${\mathscr {M}}_2$, $\ldots$, ${\mathscr {M}}_6$ with the variation of the parameter $\lambda$ from 0 to 1, Fig. 5 is used for illustration. Observing from Fig. 5, it is revealed that the rankings for ${\mathscr {M}}_1$, ${\mathscr {M}}_2$, $\ldots$, ${\mathscr {M}}_6$ remain unchange with the variation of the parameter $\lambda$ from 0 to 1. As a result, N95 is always the most desirable type of marks.

**Figure 6:**
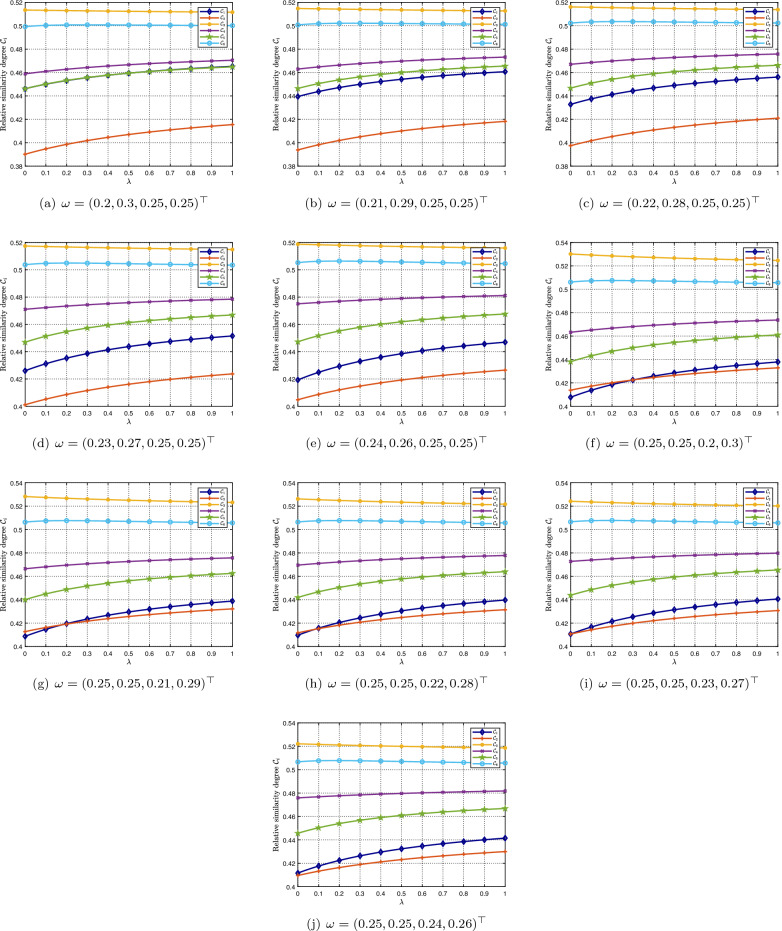
Relative similarity degrees for different values of $\lambda$ and weight vectors $\omega$.

In the above analysis, we assume that four attributes ${\mathfrak {A}}_1$–${\mathfrak {A}}_4$ have the same weight. To study the impact of the weights of attributes on the decision process, Fig. 6 is used for illustration. Observing from Fig. 6, it is revealed that although the most desirable mask type is always ${\mathscr {M}}_3$–particulate respirators (N95), the rankings of ${\mathscr {M}}_1$ and ${\mathscr {M}}_2$ may change, when changing the weights of attributes and the parameter $\lambda$.

### A medical diagnosis problem

#### *Example* 4.3

(^38^Example 4.4,^14^) Consider a medical diagnosis problem for 4 patients $\mathbb {P}=\{\mathbb {P}_1, \mathbb {P}_2, \mathbb {P}_3, \mathbb {P}_4\}$ with the symptoms ${\mathfrak {S}}=\{\text {Temperature, Headache, Stomach pain, Cough, Chest pain}\}$ represented by using IFNs, as listed in Table 8. The symptom characteristics for diagnosis ${\mathfrak {D}}=\{\text {Viral fever, Malaria, Typhoid, Stomach problem, Chest problem}\}$ are represented by using IFNs, as shown in Table 9.

**Table 8:** IFN representation of symptoms for the patients.

Patient	Temperature	Headache	Stomach pain	Cough	Chest pain
$\mathbb {P}_1$	$\langle 0.8, 0.1\rangle$	$\langle 0.6, 0.1\rangle$	$\langle 0.2, 0.8\rangle$	$\langle 0.6, 0.1\rangle$	$\langle 0.1, 0.6\rangle$
$\mathbb {P}_2$	$\langle 0.0, 0.8\rangle$	$\langle 0.4, 0.4\rangle$	$\langle 0.6, 0.1\rangle$	$\langle 0.1, 0.7\rangle$	$\langle 0.1, 0.8\rangle$
$\mathbb {P}_3$	$\langle 0.8, 0.1\rangle$	$\langle 0.8, 0.1\rangle$	$\langle 0.0, 0.6\rangle$	$\langle 0.2, 0.7\rangle$	$\langle 0.1, 0.5\rangle$
$\mathbb {P}_4$	$\langle 0.6, 0.1\rangle$	$\langle 0.5, 0.4\rangle$	$\langle 0.3, 0.4\rangle$	$\langle 0.7, 0.2\rangle$	$\langle 0.3, 0.4\rangle$

**Table 9:** IFN representation of symptom characteristics for diagnosis.

Disease	Temperature	Headache	Stomach pain	Cough	Chest pain
$\mathrm {Viral~fever~(Vf)}$	$\langle 0.4, 0.0\rangle$	$\langle 0.3, 0.5\rangle$	$\langle 0.1, 0.7\rangle$	$\langle 0.4, 0.3\rangle$	$\langle 0.1, 0.7\rangle$
Malaria (Ma)	$\langle 0.7, 0.0\rangle$	$\langle 0.2, 0.6\rangle$	$\langle 0.0, 0.9\rangle$	$\langle 0.7, 0.0\rangle$	$\langle 0.1, 0.8\rangle$
Typhoid (Ty)	$\langle 0.3, 0.3\rangle$	$\langle 0.6, 0.1\rangle$	$\langle 0.2, 0.7\rangle$	$\langle 0.2, 0.6\rangle$	$\langle 0.1, 0.9\rangle$
Stomach problem (Sp)	$\langle 0.1, 0.7\rangle$	$\langle 0.2, 0.4\rangle$	$\langle 0.8, 0.0\rangle$	$\langle 0.2, 0.7\rangle$	$\langle 0.2, 0.7\rangle$
Chest problem (Cp)	$\langle 0.1, 0.8\rangle$	$\langle 0.0, 0.8\rangle$	$\langle 0.2, 0.8\rangle$	$\langle 0.2, 0.8\rangle$	$\langle 0.8, 0.1\rangle$

By taking the weight vector $\omega$ of the 5 symptoms attributes as $\omega =(0.2, 0.2, 0.2, 0.2, 0.2)^{\top }$, based on the principle of the maximum degree of SimMs, the diagnosis results obtained by using different SimMs are listed in Table 10 with $\lambda =0.02$.

**Table 10:** Diagnostic results by using different SimMs.

Patient	Vf	Ma	Ty	Sp	Cp	Our diagnosis ($\lambda =0.02$)	Others
$\mathbb {P}_1$	0.738	**0.771**	0.742	0.435	0.393	Malaria	Malaria^12,14,15,38,44–47^ and Viral fever^48^
$\mathbb {P}_2$	0.525	0.404	0.660	**0.873**	0.582	Stomach problem	Stomach problem^12,14,15,38,44–48^
$\mathbb {P}_3$	0.669	0.600	**0.764**	0.472	0.437	Typhoid	Typhoid^14,15,38,44–48^ and Malaria^12^
$\mathbb {P}_4$	**0.728**	0.713	0.636	0.534	0.471	Viral fever	Viral fever^15,38,44,46,47^ and Malaria^12,14,45,48^

Significant values are in [bold].

**Figure 7:**
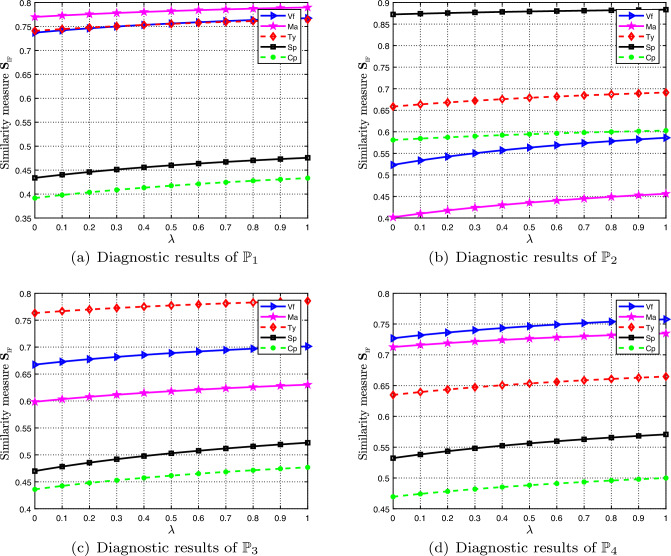
The diagnostic results of different patients for different $\lambda \in [0, 1]$.

To study the changing tendency of the diagnostic results for different patients $\mathbb {P}_1$, $\mathbb {P}_2$, $\mathbb {P}_3$, $\mathbb {P}_4$ with the variation of the parameter $\lambda$ from 0 to 1, Fig. 7 is used for illustration. Observing from Fig. 7, it is revealed that when the parameter $\lambda$ changes from 0 to 1, the diagnostic results for $\mathbb {P}_1$, $\mathbb {P}_2$, $\mathbb {P}_3$, and $\mathbb {P}_4$ are perfectly consistent with the result for $\lambda =0.02$, i.e., $\mathbb {P}_1$ suffers from ‘Malaria’, $\mathbb {P}_2$ suffers from ‘Stomach problem’, $\mathbb {P}_3$ suffers from ‘Typhoid’, and $\mathbb {P}_4$ suffers from ‘Viral fever’.

## Conclusion

To overcome the two drawbacks of Mahanta and Panda’s DisM^38^ mentioned in “The drawbacks of distance measure of Mahanta and Panda^38^”, we propose a new nonlinear parametric DisM for IFSs, which is proved to satisfy the axiomatic definition of a strict IFDisM and effectively distinguish different IFSs with high hesitancy when the parameter is sufficiently small. Moreover, we prove that the dual SimM and the induced entropy of the proposed DisM are a strict IFSimM and an IF entropy, respectively. Finally, to illustrate the effectiveness of our method, we apply our proposed DisM/SimM to the following three problems:

1. Considering an IF pattern classification problem from^14^, our proposed DisM can accurately determine to which pattern the test sample belongs. The test result shows that our proposed DisM is better than the DisMs in^23,36,38,41^;
2. To deal with an IF MADM problem on the decision making about the choice of a proper antivirus face mask for COVID-19, we propose a TOPSIS method based on our proposed strict IFSimM. The comparative analysis shows that the most desirable choice obtained by our proposed TOPSIS method with the variation of the parameter $\lambda$ from 0 to 1 is consistent with the results obtained by the TOPSIS methods in^25,35,43^. The comparative analysis also shows that the TOPSIS method in^38^ is unreasonable, because it does not consider the cost attributes for normalization;
3. We use our proposed SimM to solve an IF medical diagnosis problem. Our diagnostic results are consistent with the results in^15,38,44,46,47^.

In the paper, we had demonstrated these relative similarity degrees for different values of the parameter $\lambda$ and weights $\omega$ with the conclusion that, the ranking results in the MCDM application may change, when changing the values of the parameter $\lambda$ and weights $\omega$ of attributes. This parameter dependency becomes the drawback of the proposed method. To find a better combination of the parameter $\lambda$ and weight $\omega$ in the MCDM application becomes important, and will be a further research topic. In the future, we shall further extend our constructive methods of strict IFDisM, IFSimM and IFEM to Pythagorean fuzzy sets, q-rung orthopair fuzzy sets, T-spherical fuzzy sets, and some other interval-valued fuzzy sets.

## Data Availability

All data generated or analysed during this study are included in this published article.
